# Perinatal antibiotic exposure and risk of childhood infections: a retrospective cohort study

**DOI:** 10.1016/j.lana.2025.101264

**Published:** 2025-10-16

**Authors:** Sarah A. Coggins, William Quarshie, Robert W. Grundmeier, Di Shu, Jeffrey S. Gerber, Miren B. Dhudasia, Karen M. Puopolo, Sagori Mukhopadhyay

**Affiliations:** a Division of Neonatology, Children's Hospital of Philadelphia, Philadelphia, PA, USA; b Department of Pediatrics, University of Pennsylvania Perelman School of Medicine, Philadelphia, PA, USA; c Clinical Futures, Children's Hospital of Philadelphia, Philadelphia, PA, USA; d Department of Biomedical and Health Informatics, Children's Hospital of Philadelphia, Philadelphia, PA, USA; e Division of Infectious Diseases, Children's Hospital of Philadelphia, Philadelphia, PA, USA

**Keywords:** Antibiotics, Children, Infection

## Abstract

**Background:**

Epidemiological studies report associations between antibiotics given during pregnancy, childbirth, and infancy and subsequent risk for childhood infections. The specific role of intrapartum and neonatal antibiotic exposures is not well-described.

**Methods:**

Retrospective cohort study of healthy term infants through 6 years of age. The primary exposure was perinatal antibiotics, defined as intravenous intrapartum antibiotic administration during the admission for childbirth or administered to the infant ≤3 days after birth. The primary outcome was infection-related inpatient encounters. Adjusted multivariable-adjusted Cox proportional hazards and marginal means/rates models were used to investigate the association between exposure and outcome. A secondary analysis examined the association between early infant antibiotics administered during the first three months after birth, including perinatal antibiotics, and the outcome.

**Findings:**

Of 13,919 infants, 3936 (28%) had perinatal antibiotic exposure. 1294 (9.3%) children had 1619 inpatient encounters, of which 988 (61%) were infection-related. Infection-related inpatient encounters occurred in 265 (6.7%) children exposed to perinatal antibiotics, compared to 584 (5.8%) unexposed children (risk difference 1.2%, 95% CI 0.3–2.1%, p = 0.005). In multivariable-adjusted models, perinatal antibiotic exposure was not associated with infection-related inpatient encounters [Cox model: aHR 1.16, 95% CI 0.95, 1.51, p = 0.15; marginal rates/means model: aHR 1.22, 95% CI 0.98, 1.51, p = 0.08]. Early infant antibiotics were also not associated with the outcome.

**Interpretation:**

In this study of 13,919 newborns across 24 primary pediatric practices, perinatal or early infant antibiotic exposure was not associated with subsequent early childhood hospitalization for infectious diseases.

**Funding:**

10.13039/100009633Eunice Kennedy Shriver National Institute of Child Health and Human Development, National Institutes of Health, funding (K23 HD088753) supported this study.

**Research in context**

**Evidence before this study**

Epidemiological studies report associations between antibiotics given during pregnancy, childbirth, and infancy and subsequent risk for childhood infections. However, the specific role of intrapartum and neonatal antibiotic exposures is not well-described. We searched PubMed on December 21, 2024 for English-language articles with terms “antibiotics” AND “child” and “hospitalization”, and selected articles focusing on intrapartum and early infant antibiotic exposures. We identified three articles describing population-based analyses in Scandinavian countries. The first study identified that maternally-administered intrapartum antibiotic exposure (largely used for GBS prophylaxis) was associated with increased risk of childhood infections during 7–28 days of age (adjusted incidence rate ratio [aIRR] 1.30, 95% CI 1.10, 1.54) and at 1–2 years of age (aIRR 1.10, 95% CI 1.02, 1.18). A second study identified that maternal antibiotic exposures before or during pregnancy were associated with increased risk of childhood infection-related hospitalization (hazard ratio 1.18, 95% CI 1.17, 1.19). These associations were further present in antibiotic subtype-specific analyses, and amplified when antibiotic prescriptions were closer to the time of birth. The third study identified an association between maternal antibiotic exposures during pregnancy and the risk of childhood infections within the first year of age (aIRR 1.34, 95% CI 1.33, 1.34).

**Added value of this study**

Our analysis of almost 14,000 linked infant records from birth hospitals and pediatric practices showed that neither perinatal nor early infant antibiotic exposures were associated with an increased risk of infection-associated hospital encounters within the first six years of age. These findings were demonstrated in models assessing both the risk of any infection-related encounter, and models accounting for recurrent hospitalizations.

**Implications of all the available evidence**

In contrast to previously-published findings from Scandinavian countries, our analysis of a Philadelphia (USA) birth cohort revealed no association between perinatal or early infant antibiotic exposures and the risk of later infection-associated hospitalizations in childhood. Childhood infection-related hospitalization is likely attributable to a complex, multifactorial risk profile. Compared to the results of prior studies, our differing findings may reflect the variably defined scope of exposures and outcomes, and the populations studied in the cumulative literature on this topic. Our data suggests that concern for later childhood infectious outcomes need not serve as a barrier to the administration of indicated intrapartum antibiotics.

## Introduction

Maternally-administered intrapartum antibiotics are given prior to approximately 30% of births in the United States,^1^ most often for prevention of neonatal Group B *Streptococcus* (GBS) infection or treatment of suspected maternal intraamniotic infections. Although these intrapartum antibiotic exposures are intended to prevent life-threatening infections in mothers and newborns, early antibiotic exposures alter the neonatal gut microbiome and can result in reduced microbial diversity that is enriched with opportunistic pathogens.^2^

In epidemiological studies,^3^, ^4^, ^5^ early-life antibiotics have been associated with increased risk for subsequent infections. One potential mechanism includes disruption of the developing microbiome. This, in turn, diminishes colonization resistance, an important line of defense in preventing pathogenic blooms and gut translocation. Additionally, animal models suggest that perinatal antibiotic exposure impacts adaptive immune regulation and antiviral immunity, related to reductions in T-regulatory, natural killer cell, and dendritic cell populations in addition to impaired cytokine release.^6^^,^^7^ Such alterations may endure when they occur in the foundational early period of microbiome development.

Epidemiologic studies investigating the association between early-life antibiotics and later-life infections have examined the role of maternal antibiotics prescribed during pregnancy, and pediatric antibiotics administered for concern of infection.^3^, ^4^, ^5^ As one of the earliest forms of antibiotic exposure, administered with the explicit clinical purpose of altering the newborn's colonization,^1^ the specific contribution of perinatal antibiotic exposure is poorly understood. Studies often exclude or cannot account for perinatal antibiotics due to the need for data linkage between mother-infant dyads and across inpatient and outpatient settings. Most studies also combine high-risk and routine births. However, because preterm infants and infants with chronic congenital conditions have a higher incidence of early-life infection, antibiotic use in these births is less modifiable than prophylactic antibiotics in full-term births. Moreover, confounding by indication presents a greater challenge in high-risk infants. In this study, we used a cohort of full-term infants born without infection and chronic congenital conditions to evaluate whether perinatal antibiotics were associated with the risk of infection-related inpatient admission during the first six years of age.

## Methods

### Design and setting

This was a retrospective cohort study of infants born ≥37 weeks gestation at two perinatal centers affiliated with the University of Pennsylvania Health System in Philadelphia, PA between 2007 and 2012. The cohort consisted of mother-infant dyads delivering at a study perinatal center, where the infant then established pediatric care within 30 days of birth at any one of 24 Children's Hospital of Philadelphia (CHOP) primary care network sites. Birth hospital records and pediatric primary care records were linked using multiple identifiers, including: name, date of birth, birth hospital, infant sex, and address, with manual verification when indicated.^8^ Exclusion criteria included birth weight <2000 g or >5000 g, major chromosomal or congenital anomalies, chronic illnesses diagnosed in the neonatal period that need antibiotics as part of treatment (e.g. sickle cell disease),^9^ culture-confirmed early-onset infection within the first three days after birth, or when fewer than two primary care visits were completed. Follow-up information was collected for each subject through five years completed age (2192 days) via linked CHOP electronic medical records. A child was deemed to have left the healthcare practice if no contact was documented for >730 days. Deceased status was identified via the CHOP electronic medical record: either via direct reporting following an inpatient death within a CHOP network site, or via primary care site notification following death outside of the CHOP network. A flow diagram of study cohort derivation is shown in Supplementary Figure S1.

### Exposure

The primary exposure was perinatal antibiotics, defined as any intravenous antibiotic administered to the mother during the admission for childbirth up to five days prior to childbirth or administered to the infant within the first three days after birth. Antibiotics administered for surgical skin prophylaxis during cesarean section were excluded from this definition. Maternal intrapartum antibiotic administration (e.g., GBS prophylaxis, treatment of suspected intra-amniotic infection) is the standard of care in our study's perinatal centers, per American College of Obstetricians and Gynecologists clinical practice guidelines^10^^,^^11^ Neonatal antibiotic administration occurred in the setting of: (1) clinical illness as determined by a neonatologist, or (2) elevated sepsis risk at birth as based on perinatal risk factors.^12^ Early infant antibiotics was defined as perinatal antibiotics and/or pediatric antibiotics prescribed within the first three months after birth and was analyzed as a secondary exposure.

### Outcomes

The primary outcome was inpatient infection-related encounters that occurred at the Children's Hospital of Philadelphia, identified by pre-specified diagnostic codes (International Classification of Diseases [ICD]-9 and -10 codes) based on prior work by Miller et al.^4^

### Analytical plan and covariates

Statistical analyses included descriptive statistics to describe baseline covariates and inferences to quantify exposure-outcome associations. We utilized two multivariable-adjusted models. First, we fitted a Cox proportional hazards model relating time to any inpatient encounter for infection with exposure to perinatal antibiotics. We next leveraged a Cox proportional hazards modeling subtype to account for recurrent infection encounters.^13^ We fitted a marginal means/rates model with robust sandwich variance estimation. This model makes inferences on the means and rates of the counting process without the need for specifying dependence structures among recurrent event times within an individual.^13^^,^^14^ This robust sandwich variance is a method to estimate the covariance matrix of parameter estimates, particularly when potential correlation within clusters or subjects may exist. Infants were censored at the last recorded encounter, six years of age, or the date of death, whichever came first. We used Schoenfeld's test of residuals to assess proportionality assumptions. When covariates violated proportionality assumptions, the models were run stratified by those covariates. Based on the residual plots, test output, and changes in estimates for the primary exposure, we present the estimates from the original model and the estimates from the stratified models in the supplementary results.

Models were adjusted for the following baseline covariates: birth year and season, maternal age, maternal parity, maternal race (self-reported, acknowledged as a social construct and included given potential association with social determinants of health and healthcare access), mode of delivery, maternal GBS colonization status, maternal obesity, maternal asthma, chorioamnionitis, infant birth weight, infant sex, child insurance status, and variables linked to the child's home address via the American Community Survey (education and income levels) (Supplementary Table S1). These variables were included as potential confounders of the risk of intrapartum and/or early-onset neonatal infection, the risk of childhood infection, as well as social determinants of health potentially influencing both perinatal and childhood health outcomes.^3^, ^4^, ^5^^,^^15^ A directed acyclic graph to depict these relationships is shown in Supplementary Figure S1. When modeling the secondary exposure, vaccination status in infancy (defined as receipt of pneumococcal and/or *H. influenzae* type B vaccination within the first three months after birth) and breastfeeding exposure within the first three months after birth were included as additional covariates. These variables were included as potential negative confounders of childhood infection risk given the protective effects of active and passive immunity.^15^^,^^16^ All analyses were performed using SAS statistical software (version 9.4, Cary, NC, USA).

### Ethics approval

This study was approved with a waiver of informed consent by the Institutional Review Boards at Children's Hospital of Philadelphia (# 16-012944, 9/30/2016).

### Role of the funding source

This study was funded by the National Institutes for Child Health and Human Development of the National Institutes of Health (K23 HD088753 to SM). The funder had no role in study design, data collection, data analysis, interpretation, or writing of the manuscript.

## Results

There were 15,986 infants with linked hospital records available for analysis. After applying exclusion criteria, 2067 infants were excluded, leaving 13,919 infants who composed the final study cohort and were included in the analysis (Supplementary Figure S2). Among all infants in the study cohort, 3936 (28%) infants had exposure to perinatal antibiotics. Maternal antibiotic administration was the most common form of perinatal antibiotic exposure, accounting for 85% (3338/3936) exposed infants. Neonatal antibiotics were administered to 28% (1121/3936) of infants with perinatal antibiotic exposures (Supplementary Table S2). Clinical and demographic characteristics of the cohort are presented in Table 1. Perinatal antibiotic exposure was significantly associated with lower maternal age (26 vs. 29 years, p < 0.001) and maternal parity (p < 0.001). Maternal race/ethnicity differed significantly by perinatal antibiotic exposure status (p > 0.001): Black race was associated with a higher proportion of antibiotic exposure (65.2%) compared to non-exposure (54.6%), while the opposite was identified for White individuals (21.6% vs 28.4%, respectively). Lower mean birth gestation (39.4, SD 1.1 vs. 39.6 SD 1.2) and male sex (53.4% vs. 49.8%) were associated with perinatal antibiotics.

**Table 1:** Demographic and clinical characteristics of the study population, 2007–2012.

Characteristic^a^^b^	Overall cohort N = 13,919	Any perinatal antibiotics N = 3936	No perinatal antibiotics N = 9983	p-value
Maternal age (median, IQR)	28 (23, 33)	26 (21, 32)	29 (24, 33)	<0.001
Maternal race/ethnicity				<0.001
Black	8016 (57.6%)	2568 (65.2%)	5448 (54.6%)	
White	3686 (26.5%)	850 (21.6%)	2836 (28.4%)	
Asian	1000 (7.2%)	217 (5.5%)	783 (7.8%)	
Hispanic	472 (3.4%)	109 (2.8%)	363 (3.6%)	
Other/Unknown	745 (5.4%)	192 (4.9%)	553 (5.5%)	
Maternal parity				<0.001
1	6327 (45.5%)	2248 (57.1%)	4079 (40.9%)	
≥2	7461 (53.6%)	1663 (42.3%)	5798 (58.1%)	
Cesarean section	4291 (30.8%)	1052 (26.7%)	3239 (32.4%)	<0.001
Maternal chorioamnionitis	774 (5.6%)	669 (17.0%)	105 (1.1%)	<0.001
Maternal asthma	1897 (13.6%)	605 (15.4%)	1292 (12.9%)	<0.001
Maternal Group B *Streptococcus* status				<0.001
Negative	9248 (66.4%)	947 (24.1%)	8301 (83.2%)	
Positive	3889 (27.9%)	2861 (72.7%)	1028 (10.3%)	
Unknown	782 (5.6%)	128 (3.3%)	654 (6.6%)	
Maternal body mass index (kg/m^2^)				<0.001
<18.5	23 (0.2%)	2 (0.1%)	21 (0.2%)	
18.5–<25.0	1847 (13.3%)	450 (11.4%)	1397 (14.0%)	
25.0–<30.0	4943 (35.5%)	1268 (32.2%)	3675 (36.8%)	
≥30.0	7091 (50.9%)	2215 (56.3%)	4876 (48.8%)	
Male sex (child)	7071 (50.8%)	2102 (53.4%)	4969 (49.8%)	<0.001
Gestational age, mean (SD)	39.4 (1.1)	39.4 (1.1)	39.6 (1.2)	<0.001
Birth weight, mean (SD)	3318 (456)	3309 (457)	3340 (453)	<0.001
Birth weight for gestation z score, mean (SD)^c^	−0.17 (0.86)	−0.16 (0.86)	−0.19 (0.85)	0.12
Insurance at first pediatric visit				<0.001
Public	6090 (43.8)	1844 (46.9)	4246 (42.5)	
Private	7410 (53.2)	1972 (50.1)	5438 (54.5)	
Self	419 (3.0)	120 (3.1)	299 (3.0)	
**Tract link census data**^d^				
Median household income quartiles, US dollars				<0.001
≤27,800	3456 (24.8%)	1084 (27.5%)	2372 (23.8%)	
27,801–≤39,610	3449 (24.8%)	1011 (25.7%)	2438 (24.4%)	
39,611–≤62,315	3456 (24.8%)	975 (24.8%)	2481 (24.9%)	
≥62,316	3469 (24.9%)	839 (21.3%)	2630 (26.3%)	
Proportion of residents with less than high school education, median (IQR)	0.17 (0.08, 0.27)	0.18 (0.09, 0.28)	0.17 (0.08, 0.27)	<0.001
Children with infection-related inpatient encounter	849 (6.1%)	265 (6.7%)	584 (5.8%)	0.05
Single episode	758 (89.3%)	239 (90.2%)	519 (88.9%)	
More than one episode	91 (10.7%)	26 (9.8%)	65 (11.1%)	
**Infants included in analysis for early antibiotics (3-month)**	**13,529**	**3833**	**9696**	
Completed vaccine schedule	12,253 (90.6%)	3467 (90.5%)	8786 (90.6%)	0.77
Breastfeeding at 3 months of age	5976 (44.2%)	1558 (40.6%)	4418 (45.6%)	<0.001
Exposure to antibacterials in first 3 months after discharge from birth hospitalization	401 (3.0%)	105 (2.7%)	296 (3.1%)	0.33

a Any perinatal antibiotics defined as any intravenous antibiotic administered to the mother during the admission for childbirth (up to five days prior to childbirth) or administered to the infant within the first three days after birth.

b Missing values for characteristics were <1% of the total cohort. Information missing for the following variables among exposed vs. unexposed: maternal age (1, 3); maternal race (1,3); parity (25, 106); maternal BMI (1, 14); median household income (27, 62); proportion of residents with less than high school education (17, 46).

c Sex-specific BW for GA z-scores derived from Fenton growth charts.

d Proportion of residents with less than high school education and median household income are derived from American Community Survey (ACS) 2009–2012 data including ACS tract-level data (detailed definition in Supplementary Table S1). IQR, inter-quartile range; SD, standard deviation; US, United States.

**Fig. 1:**
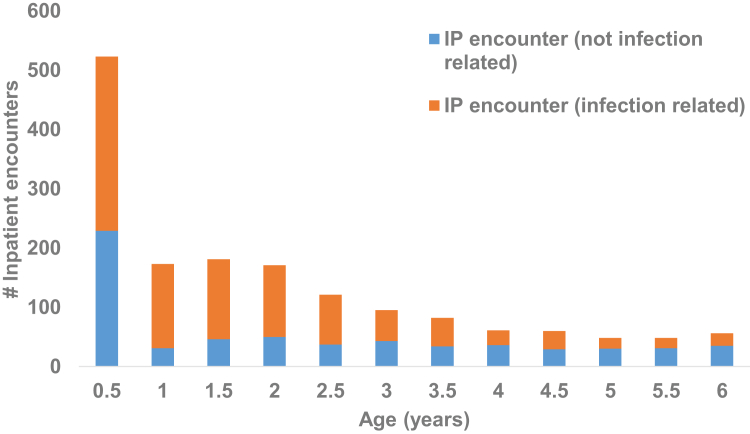
Stacked bar chart reflecting the frequency of infection-related inpatient encounters (orange) among non-infection-related inpatient encounters (blue), by age.

There were 13 deaths (<0.1%) during the study period, three among exposed infants and ten among infants not exposed to perinatal antibiotics. In the last year of follow-up, 10,201 infants were retained in the cohort (73%). During the study period 1294 (9.3%) children had 1619 inpatient encounters. Among these, 988 (61%) were infection-related and occurred among 849 children; 758 children (89.3%) had one infection-related inpatient encounter, while 91 infants (10.7%) had two or more infection-related encounters (Table 1) Of the 988 infection-related inpatient encounters, 436 (44.1%) occurred in the first year after birth (Fig. 1). The most common diagnosis codes associated with inpatient encounters included those for acute respiratory infections, viral infections, and urinary tract infection (Supplementary Table S3).

Infection-related inpatient encounters occurred in 265 (6.7%) children exposed to perinatal antibiotics, compared to 584 (5.8%) children without such exposure (risk difference 1.2%, 95% CI 0.3–2.1%, p = 0.005). In bivariable analysis, perinatal antibiotics had a hazard ratio of 1.14 (95% CI 0.98, 1.32, p = 0.08). The unadjusted hazard ratio for other covariates is shown in Supplementary Table S4. In the multivariable-adjusted Cox proportional hazards modeling showed that perinatal antibiotic exposure was not associated with infection-related inpatient encounter [adjusted hazard ratio (aHR) of 1.16 (95% CI 0.95, 1.51; p = 0.15)]. Recurrent event modeling yielded similar findings, with aHR 1.21 (95% CI 0.98, 1.51; p = 0.08, Table 2). Estimates for the exposure in multivariable models stratified by covariates that violated the proportionality assumption were not different (Supplementary Table S5).

**Table 2:** Multivariable models for antibiotic exposure with outcomes.

	Adjusted hazard ratio	95% CI	p-value
**Primary model**^a^			
**Cox proportional hazards**			
No antibiotics^c^	Ref	–	–
Perinatal antibiotics only^d^	1.16	0.95, 1.51	0.15
**Recurrent event analysis**			
No antibiotics^c^	Ref	–	–
Perinatal antibiotics only^d^	1.22	0.98, 1.51	0.08
**Secondary model**^b^			
**Cox proportional hazards**			
No antibiotics^c^	Ref	–	–
Perinatal antibiotics only^d^	1.05	0.88, 1.25	0.59
Pediatric antibiotics in first 3 months only^e^	1.07	0.63, 1.82	0.81
Both perinatal and pediatric antibiotics in first 3 months^f^	0.59	0.19, 1.84	0.36
**Recurrent event analysis**			
No antibiotics^c^	Ref	–	–
Perinatal antibiotics only^d^	1.14	0.93, 1.39	0.22
Pediatric antibiotics in first 3 months only^e^	1.27	0.73, 2.23	0.40
Both perinatal and pediatric antibiotics in first 3 months^f^	0.87	0.24, 3.17	0.83

a Cox proportional hazards and marginal means/rates (recurrent analysis) models for the primary exposure, defined as any intravenous antibiotic administered to the mother during the admission for childbirth up to five days prior to childbirth or administered to the infant within the first three days after birth. Models adjusted for birth year and season, maternal age, maternal parity, maternal race, mode of delivery, maternal GBS colonization status, maternal obesity, maternal asthma, chorioamnionitis, infant birth weight, infant sex, child insurance status, and education and income levels.

b Cox proportional hazards and marginal means/rates (recurrent analysis) models for the secondary exposure, defined as perinatal antibiotics and/or pediatric antibiotics prescribed within the first three months after birth. Models adjusted for same variables as the primary model, plus vaccination status in infancy and breastfeeding status.

c No maternal nor neonatal antibiotic exposures near the time of birth.

d Receipt of an intravenous antibiotic administered to the mother during the admission for childbirth and within five days of delivery, or administered to the infant within the first three days after birth.

e Any infant antibiotic exposure during the first three months after birth.

f Any perinatal or pediatric antibiotic exposures during the first three months after birth, as defined above in (2) and (3).

Similar to the primary exposure, the secondary exposure of early infant antibiotics in the first three months after birth was not associated with infection-related inpatient encounters (Table 2).

## Discussion

Within this retrospective longitudinal birth cohort, we did not identify significant associations between perinatal or early infant antibiotic exposures and later risk of infection-related childhood inpatient admissions. Secondary analyses accounting for antibiotic exposures within the first three months after birth yielded similar conclusions.

Childhood infections are common and are thus frequent drivers of pediatric inpatient hospitalizations. In 2016, infectious conditions accounted for nine of the 20 most common diagnoses prompting pediatric inpatient admissions captured within the KID database (with bronchiolitis, pneumonia, and cellulitis ranking first, second, and fifth in frequency, respectively).^17^ In a Danish prospective cohort study that followed children from birth to three years of age, participants had a median of 14 infectious episodes (with wide variability in range, from two to 43 episodes).^15^ Risk factor profiling within this cohort identified environmental drivers of childhood infections, but such factors explained only 8% of the observed infections and led study authors to posit unmeasured host factors as significant additional drivers of infection. In another population-based cohort of births in Western Australia, risk of infection-related childhood inpatient hospitalization was significantly associated with successive decreases in gestational age, birth weight, and birth length.^18^

Childhood infection-related hospitalization is likely attributable to a complex, multifactorial risk profile. However, in recognition of the relationship between antibiotic exposure, altered microbiota, and infectious sequelae,^19^, ^20^, ^21^, ^22^, ^23^, ^24^, ^25^ there has been great interest in evaluating the potential contribution of perinatal and early-life antibiotic exposure upon risk of childhood infections. In contrast to our findings, three prior studies from Scandinavian countries have identified associations between early-life antibiotic exposure and childhood infections. A population-based study of births in Northern Finland found that exposure to maternally-administered intrapartum antibiotics (largely used for GBS prophylaxis) was associated with increased risk of childhood infections during 7–28 days of age and at 1–2 years of age.^3^ However, this analysis was not specific to infection-related inpatient admissions, but also included outpatient and emergency room encounters. Another population-based study of Danish births identified that maternal antibiotic exposures before or during pregnancy were associated with increased risk of childhood infection-related hospitalization.^4^ These associations were further present in antibiotic subtype-specific analyses, and amplified when antibiotic prescriptions were closer to the time of birth. Of note, Group B *Streptococcus* screening was not routinely performed in this cohort, and intrapartum antibiotic administration for GBS prophylaxis was therefore unlikely. Finally, a recent population-based cohort study in Sweden identified an association between maternal antibiotic exposures during pregnancy and the risk of childhood infections within the first year of age.^5^ However, outcomes included infections diagnosed in both outpatient and inpatient settings, and the strength of association was attenuated following sibling analyses accounting for familial factors.

Our findings differed from those in the studies described above, which may be attributable to multiple factors. First, the scope of exposures (perinatal antibiotic exposure) and outcomes (childhood infections) were variably defined between these prior studies and ours, precluding direct comparison of the results. Studies focused specifically on intrapartum and early neonatal antibiotic exposures, like ours, remain rare. Because our data sources were linked electronic medical records, we could utilize several covariates in our models that were not used in prior studies (e.g., neighborhood social factors, vaccination status, breastfeeding status), which may have influenced outcome estimates towards the null results we report. Lastly, there are several differences in the population of our study, both in geography and due to the exclusion of children with chronic congenital diseases.

Our study was strengthened by using linked birth and childhood medical records, which allowed follow-up through six years of age. As a result, we had access to highly granular data describing the circumstances of birth, perinatal antibiotic exposure, and the timing of and diagnoses associated with childhood inpatient encounters. Perinatal antibiotics are documented as administered and are less prone to bias from variable compliance with prescribed outpatient antibiotics. Finally, our use of time-to-event analyses facilitated efficient use of the full data set by accounting for recurrent infection-attributable inpatient encounters.

We also acknowledge limitations. Our study reflected a full-term healthy birth cohort from two hospitals in Philadelphia, United States, and our results may not be generalizable to other populations or settings. We adjusted for multiple covariates to account for potential confounding between the outcome and exposures. While alternative approaches (e.g., propensity score matching) can improve baseline covariate balance, they do not eliminate residual confounding and we did not utilize such approaches for this reason. Death was a rare outcome unlikely to compete with the main study outcome, and for this reason we did not perform competing outcomes analyses. We censored children from the study if they had no contact with the healthcare system for >730 days. However, the exact timing of exit from the cohort is uncertain, and this is a limitation of these real-world data. We acknowledge that infection-related inpatient encounters occurring outside of our pediatric health network, while likely uncommon, would not have been reflected in our results. Our study's focus was solely on infection-related inpatient encounters, as the most serious outcome potentially associated with perinatal antibiotic exposures. However, different relationships may exist between perinatal antibiotic exposures and other types of infection encounters (e.g., those occurring in primary care offices, urgent care centers, and/or emergency departments). Lastly, an altered microbiome has been proposed as a potential mechanism linking early-life antibiotic use and later infection risk. In experimental models, perinatal equivalent antibiotics increase the risk of morbidity with subsequent infection via alterations in the microbiome.^26^ In clinical studies, perinatal antibiotics are associated with enduring changes to the microbiome,^27^ and altered microbiomes are observed prior to serious bacterial infections.^22^ As microbiome data were not available in this retrospective study, we cannot determine whether the lack of an observed association between perinatal antibiotics and the risk of childhood infection also reflects a lack of relationship between these factors and the microbiome. It is possible that even if microbiome changes occurred, they were not severe enough or did not occur frequently enough to result in a measurable increase in severe infections. It is also possible that the effect of any perinatal antibiotic-induced dysbiosis upon later infection risk was mitigated by other protective factors in childhood, including breastfeeding, vaccination, and dietary contributions.

### Conclusion

Perinatal antibiotic exposure, either alone or in conjunction with pediatric antibiotic exposure in the first three months after birth, was not associated with increased hazards of childhood infectious diseases requiring inpatient hospitalization in the first six years after birth.

## Contributors

SAC contributed to the investigation and wrote the original draft of the manuscript.

WQ and MD contributed to data curation, formal analysis, interpretation of results, and wrote the manuscript–review and editing.

DS and RWG contributed to conceptualization, formal analysis, investigation, and wrote the manuscript–review and editing.

JSG and KMP contributed to supervision, conceptualization, investigation, and wrote the manuscript–review and editing.

SM contributed to funding acquisition, conceptualization, methodology, formal analysis, investigation, wrote the manuscript–review and editing, and directly accessed and verified the data.

SM and WQ authors confirm full access to study data.

All authors accept responsibility to submit for publication.

## Data sharing statement

The data analyzed in this study can be made available on request to the corresponding author with appropriate institutional approvals.

## AI use statement

We did not employ generative artificial intelligence for any aspect of this study, including data collection, analysis, or writing of the manuscript.

## Declaration of interests

KMP reports relationships with the National Institutes of Health, UpToDate, Inc., *Clinics in Perinatology, Pediatrics*, and the American Academy of Pediatrics, all of which are unrelated to the conduct of this study.
