# Supplementary material for: Perinatal antibiotic exposure and risk of childhood infections: a retrospective cohort study
Source: Lancet Reg Health Am. 2025 Oct 16;52:101264. doi: 10.1016/j.lana.2025.101264 (PMC12553004; doi:10.1016/j.lana.2025.101264)
Supplement: Supplementary Files [file mmc1.docx]

**Supplementary Figure 1: Directed Acyclic Graph**


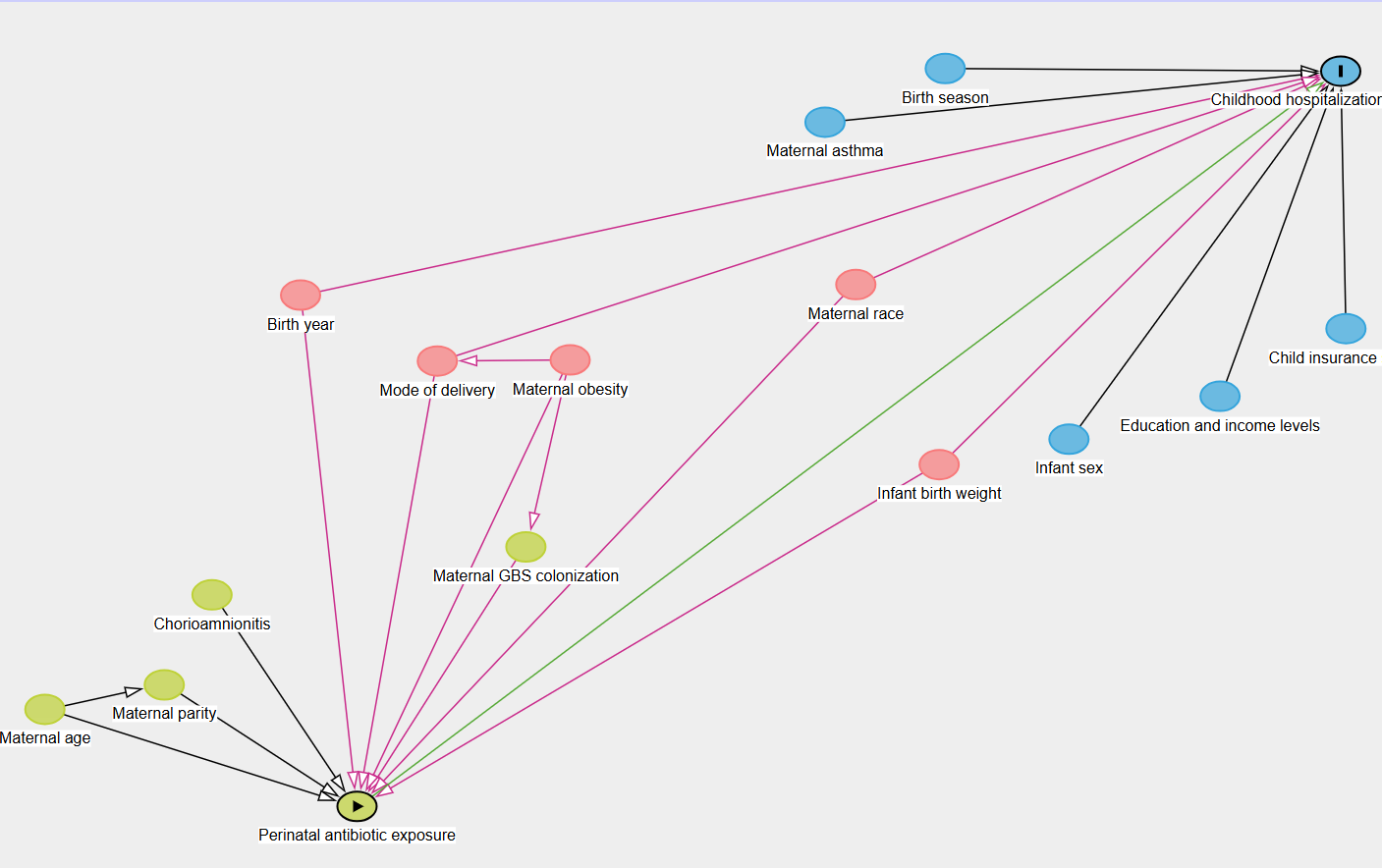
**Figure Legend:** The green circle with an arrow represents the exposure of interest, while the blue circle with a bar indicates the primary outcome. Plain green circles depict the ancestor of the exposure, blue plain circles show the ancestor of the outcome, and red circles represent the ancestors of both the exposure and outcome. The red lines indicate biasing pathways that should be included in a minimal model, and the green pathway is the causal pathway. Figure made using [DAGitty - drawing and analyzing causal diagrams (DAGs)](https://www.dagitty.net/).

**Supplementary Figure 2. Study cohort derivation**


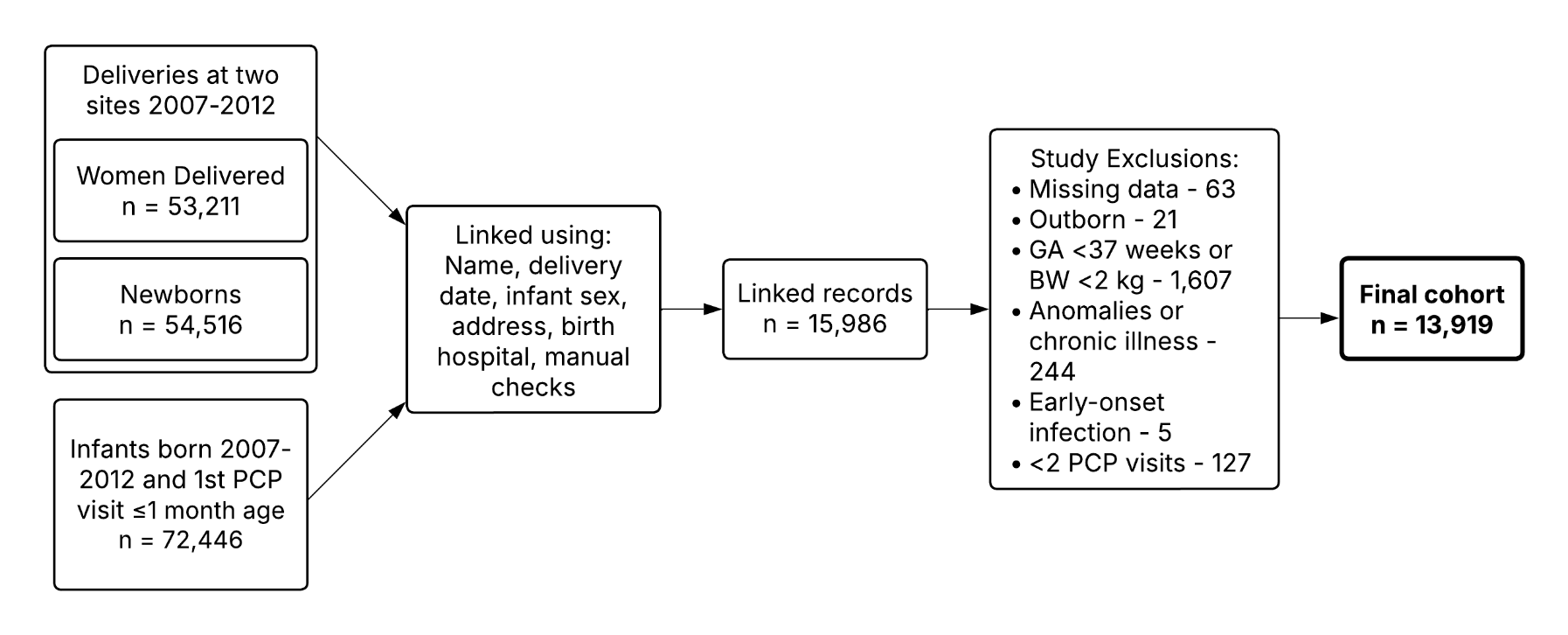


**Figure legend:** Flow diagram of study cohort derivation. PCP: primary care provider; GA: gestational age; BW: birth weight

**Supplementary Table 1. Definition and derivation of covariates**

| **Variable** | **Source** | **Definition** |
| --- | --- | --- |
| Perinatal antibiotics | Medical record/pharmacy data | All intravenous and intramuscular antibiotics administered to the mother during the hospital admission leading to delivery (upper margin 5 days prior to delivery) and to the neonate in the first 3 days after birth excluding antibiotics administered as part of surgical skin prophylaxis in cesarean section. |
| Maternal age | Labor room delivery summary | Age of the mother calculated from her date of birth and age at the time of childbirth admission |
| Maternal race/ethnicity | Maternal medical record and infant’s birth certificate | Reported value at the time of admission leading to delivery. Categorical variable as White/Non-Hispanic, Black/Non-Hispanic, Asian, Hispanic, and Other/Unknown. |
| Maternal body mass index | Calculated from maternal weight and height | Maternal weight and height from maternal medical chart as reported at admission for delivery or from infant’s birth certificate. |
| Parity | Labor room delivery summary | Recorded after delivery of infant. Categorized as 1,2,3 and more than 3. |
| Group B streptococcus screening status | Labor room delivery summary | Recorded status as known at the time of delivery. Categorized as positive, negative, or unknown at the time of birth. |
| Chorioamnionitis | Labor room delivery summary, maternal and infant admission diagnostic codes | Maternal ICD-9 diagnosis codes 658.41, 658.43, 658.4 or infant ICD9 diagnosis code 762.7, or text extraction from labor room summary notable for maternal chorioamnionitis or fever. |
| Maternal asthma | Labor room delivery summary, maternal admission diagnostic codes | Maternal ICD-9 diagnosis codes 493, 493.02, 493.2, 493.22, 493.81, 493.9, 493.91, 493.92, or labor room summary mentioning history of asthma. |

**Supplementary Table 1 (Continued). Definition of covariates**

| **Variable** | **Source** | **Definition and variable characteristic in the model** |
| --- | --- | --- |
| Birth season | Derived from date of birth | Categorized as four seasons: March – May; June – August; September-November; December -February |
| Insurance | Pediatric record | Payor at first pediatric visit. Categorized as public, private and self-pay |
| Variables related to residence | American Community Survey (ACS) 2009-2012 | Children were linked to the ACS tract level data Using the Federal Information Processing Series codes associated with the child’s earliest residential address documented in the pediatric record. |
| Proportion with less than high school education |  | Percent of population from tract level census data whose level of education was less than high school. Continuous variable. |
| Median household income quartile |  | Median household income (US dollars) from tract level census data divided into quartiles. Categorical variable as ≤27800, 27801 to ≤39610, 39611 to ≤62315, and ≥62316 |
| Birth weight Z scores | [www.peditools.com](http://www.peditools.com) | Z-scores obtained by inputting sex, birth weight, and gestational age obtained from labor room delivery summary into the Fenton based calculator. Continuous variable. |
| Completed vaccine schedule | Pediatric immunization records | Children with receipt of pneumococcal and/or *H. influenzae* type B vaccination within the first three months after birth were deemed as completed vaccine schedule during that period |
| Breastfeeding, 3 months | Pediatric medical records | Text string following “Diet” field in all pediatric progress notes were extracted and manually reviewed for presence of breastfeeding. Review started at the progress note closest to the 6-month visit. No breastfeeding was assigned when no note between 0-6 months mentioned breastfeeding. Breastfeeding duration was determined at the age of the progress note that last mentioned any breast feeding. Breastfeeding at 3 months was then derived as a binary variable from the data. |

**Supplementary Table 2: Source of antibiotics in perinatal antibiotic exposure, 2007-2012**

| **Source** | **Perinatal antibiotics**^1^  **N=3936** |
| --- | --- |
| Maternal antibiotics only | 2815 (71.5%) |
| Neonatal antibiotics only | 598 (15.2%) |
| Both maternal and neonatal antibiotics | 523 (13.3%) |

Footnote: ^1^Any perinatal antibiotics defined as any intravenous antibiotic administered to the mother during the admission for childbirth (up to five days prior to childbirth) or administered to the infant within the first three days after birth.

**Supplementary Table 3: Most common infection-related ICD-9/10 codes associated with inpatient encounters,** **2007-2012**

| **ICD-9/10 Code** | **Description** | **Count** |
| --- | --- | --- |
| 466.19 | Acute bronchiolitis due to other infectious organisms | 236 |
| 465.9 | Acute upper respiratory infections of unspecified site | 223 |
| 486 | Pneumonia, organism unspecified | 133 |
| 079.3 | Rhinovirus infection in conditions classified elsewhere and of unspecified site | 129 |
| 466.11 | Acute bronchiolitis due to respiratory syncytial virus (RSV) | 122 |
| 382.9 | Unspecified otitis media | 118 |
| 079.99 | Unspecified viral infection | 91 |
| 079.0 | Adenovirus infection in conditions classified elsewhere and of unspecified site | 57 |
| 079.89 | Other specified viral infection | 48 |
| 599.0 | Urinary tract infection, site not specified | 44 |

**Supplementary Table 4: Bivariable analysis of covariate association with inpatient infection-related encounters**

| **Covariate** | **Hazard Ratio** | **Lower CL^1^** | **Upper CL** |
| --- | --- | --- | --- |
| Perinatal antibiotics | 1.14 | 0.98 | 1.32 |
| Maternal age in years | 0.95 | 0.94 | 0.96 |
| Maternal race & ethnicity |  |  |  |
| White/Non-Hispanic | Reference |  |  |
| Black/Non-Hispanic | 2.19 | 1.81 | 2.66 |
| Asian | 1.28 | 0.89 | 1.83 |
| Hispanic | 1.15 | 0.70 | 1.89 |
| Other/Unknown | 1.25 | 0.84 | 1.88 |
| Primipara | Reference |  |  |
| 2 | 1.16 | 0.98 | 1.37 |
| 3 | 1.42 | 1.16 | 1.74 |
| 4 | 1.52 | 1.15 | 2.00 |
| >4 | 1.64 | 1.20 | 2.23 |
| Cesarean birth | 0.99 | 0.85 | 1.14 |
| Maternal asthma | 1.88 | 1.60 | 2.22 |
| GBS status |  |  |  |
| Negative | Reference |  |  |
| Positive | 1.09 | 0.94 | 1.27 |
| Unknown | 1.31 | 1.00 | 1.72 |
| Maternal obesity | 1.37 | 1.11 | 1.70 |
| Male sex | 1.28 | 1.11 | 1.47 |
| Birthweight (per 500 grams) | 0.87 | 0.80 | 0.93 |
| Infants Insurance |  |  |  |
| Public | Reference |  |  |
| Private | 0.85 | 0.74 | 0.98 |
| Self | 1.15 | 0.79 | 1.66 |
| Median Household income (in US dollars |  |  |  |
| ≤27,800 | 1.02 | 0.86 | 1.22 |
| 27,801 to ≤39,610 | 0.81 | 0.67 | 0.98 |
| 39,611 to ≤62,315 | 0.47 | 0.38 | 0.59 |
| ≥62,316 | Reference |  |  |
| Proportion of residents with less than high school education^1^ | 3.56 | 2.26 | 5.63 |

**Footnote:** ^1^ CL: confidence limit. ^2^Proportion of residents with less than high school education and median household income are derived from American Community Survey 2009-2012.

**Supplementary Table 5: Estimates for the exposure in multivariable models stratified by covariates violating the proportionality assumption**

|  | **Adjusted Hazard Ratio^3^** | **95% CI** | **p-value** |
| --- | --- | --- | --- |
| **Cox Proportional Hazards stratified by birth year and season** |  |  |  |
| No antibiotics^1^ | Ref | -- | -- |
| Perinatal antibiotics only^2^ | 1.16 | 0.96, 1.41 | 0.12 |
| **Recurrent Event Analysis stratified by birth season, maternal asthma and parity** |  |  |  |
| No antibiotics^1^ | Ref | -- | -- |
| Perinatal antibiotics only^2^ | 1.22 | 0.98, 1.51 | 0.08 |

^1^ No maternal nor neonatal antibiotic exposures near the time of birth

^2^ Receipt of an intravenous antibiotic administered to the mother during the admission for childbirth and within five days of delivery, or administered to the infant within the first three days after birth.

^3^ Models adjusted for birth year and season, maternal age, maternal parity, maternal race, mode of delivery, maternal GBS colonization status, maternal obesity, maternal asthma, chorioamnionitis, infant birth weight, infant sex, child insurance status, and education and income levels.
